# Prospects for Complete DNA Barcode Coverage of the New Zealand Insect Fauna

**DOI:** 10.1002/snz2.70078

**Published:** 2026-08-14

**Authors:** Thomas R. Buckley, Andrew Dopheide, Darren Ward, Manpreet Dhami

**Affiliations:** ^1^ Bioeconomy Science Institute Auckland New Zealand; ^2^ School of Biological Sciences The University of Auckland Auckland New Zealand; ^3^ Bioeconomy Science Institute Lincoln New Zealand

**Keywords:** barcode index numbers, biodiversity, diagnostics, identification, mitochondrial DNA, operational taxonomic unit, taxonomic bias

## Abstract

The accurate identification of insects supports conservation, biosecurity and ecological monitoring. However, morphological identification is constrained by taxonomic complexity, undescribed diversity, and limited expertise. DNA barcoding can alleviate some of these challenges and complement morphological identification, if reference data are comprehensive and reliable. We assessed the taxonomic, geographic and elevational coverage of New Zealand insect DNA barcodes using taxonomic names from the New Zealand Organisms Register, published estimates of undescribed species richness, and DNA barcode metadata. Only 18% of described native and exotic insect species currently have DNA barcodes, with higher coverage for exotic (59%) than native species (12%). DNA barcode coverage varies from complete in several single‐species orders to very low in hyperdiverse orders such as Coleoptera (7%) and Diptera (5%), while moderately diverse aquatic orders reach 31%–91% coverage. Low elevation and populated regions are overrepresented in the DNA barcode data, and many species identifications are based on bioinformatic approaches rather than taxonomic or diagnostic expertise. Despite these biases, we propose that a complete national DNA barcode library for described insect species is highly achievable given access to well‐curated insect collections and species name databases for tracking progress. Complete DNA barcoding of undescribed species will be more challenging and will require targeting underrepresented taxa, geographic regions and elevations. Sampling will need to incorporate field surveys with bulk specimen collection, environmental DNA, museomic approaches and expert‐verified identifications. Completing this library will strengthen New Zealand's regional and national‐scale biodiversity monitoring using environmental DNA, with important contributions to biosecurity and conservation outcomes.

## 1 Introduction

The identification of insect species is critical for maintaining an effective biosecurity system, conserving biodiversity and monitoring environmental change. The New Zealand Pest Register tracks high‐priority biosecurity threats and contains nearly 15,000 species, with over half of them being insects. Similarly, of the 240 species listed under the New Zealand Biosecurity Notifiable Organisms Order 2016—organisms recognised as posing significant threats to the country's environment or economy and subject to legally mandated reporting—85 species (35%) are insects. Therefore, the accurate and rapid identification of insects is critical to New Zealand biosecurity. In a conservation context, the New Zealand Threat Classification System (Rolfe et al. 2022) lists 4316 species, including approximately 1400 insect species (Barnsley 2021), and accurate identification of these taxa is fundamental to assessing their rarity and extinction risk. Insects also play critical roles in ecosystem services such as pollination (Newstrom and Robertson 2005), pest suppression (Malone et al. 2017) and nutrient recycling via decomposition (Meurk et al. 2013), yet monitoring these processes is partly dependent on accurate identification of insect species. Furthermore, much fundamental entomological research is ultimately based on accurate species identifications.

Insect species are often extremely difficult to identify for several reasons (van Klink et al. 2024). First, many described species have not undergone modern taxonomic study and therefore lack the dichotomous keys, descriptions and images required to identify species using morphological characters. Second, many closely related species are inherently difficult to distinguish, requiring taxon‐specific expertise and time‐consuming methods such as microscopy and dissection. Such identification methods can be hard to scale, which is problematic when large numbers of specimens are present and rapid decision‐making is required in the biosecurity context. Third, even when a taxon has been subjected to modern taxonomic revision, identification often remains reliant on highly specialised diagnostic and taxonomic capability, which is not always available. Fourth, some groups are taxonomically unstable, and reliable identification requires detailed knowledge of the taxonomic literature, which can be difficult to access and interpret for non‐specialists. Finally, large numbers of New Zealand insect species are yet to be described, and so obtaining species‐level identifications is not possible. Many of these problems can be alleviated through modern taxonomic revision but the expertise to achieve this (for the New Zealand insects and more generally) is already in short supply and declining further (Lester et al. 2014; Buckley 2025; Leschen et al. 2026).

For over 20 years, DNA barcoding has been promoted as a method for identifying species when morphological diagnostic capability and taxonomic resources are not available (Hebert et al. 2003). DNA barcoding (*sensu* Hebert et al. 2003) is based on a 658 base pair region of the mitochondrially encoded cytochrome c oxidase subunit I gene. DNA barcoding can assign specimens to molecular operational taxonomic units (MOTUs) or barcode index numbers (BINs), which are clusters of DNA barcodes that can be used as proxies for species under certain assumptions (Ratnasingham and Hebert 2013). If a described species has existing DNA barcodes, then unknown specimens can be matched to that species via comparison of the DNA sequences (Hebert et al. 2003). DNA barcoding has some well‐known limitations due to the biology of the mitochondrial genome, the most significant being that a species will not necessarily be monophyletic for mtDNA haplotypes due to introgression and incomplete lineage sorting (Moore 1995; Wheeler 2005; Will et al. 2005). While identification accuracy improves as the taxonomic coverage of DNA barcode databases increases (Fujisawa and Imai 2026), these biological limitations are inherent to the method and must be kept in mind when interpreting DNA barcoding analyses (Meyer and Paulay 2005; Zenker et al. 2024). Nevertheless, DNA barcoding has become a standard approach for species identification and diagnostics, especially in biosecurity, but is also increasingly used for a wide range of ecological and environmental studies. It can readily match individuals to the same species despite differences in sex or life stages (e.g., adults versus larvae) or when only small sample fragments are present (e.g., leg, head and gut contents).

Further demand for DNA barcoding comes from the surge of environmental DNA (eDNA)‐based tools for biodiversity monitoring and species detection (Holdaway et al. 2017; Wilkinson et al. 2024). The raw data from an eDNA study typically consist of short DNA sequence reads from a targeted barcode region from the various organisms represented in the input sample, which are processed into sequence‐delineated taxonomic units, which are then assigned taxonomic identities via comparison with a DNA barcode reference library—i.e., a curated database of DNA barcode sequences linked to validated taxonomic identifications (Ratnasingham and Hebert 2007). However, these reference libraries currently lack coverage of most insect species (Blackman et al. 2024; Ward 2024). This in turn limits taxonomic identifications of many sequences to relatively high taxonomic levels (e.g., Order or Family), meaning that the biological insights that can be obtained are severely restricted. DNA barcode data from accurately identified specimens offer a way of populating reference libraries, allowing eDNA sequences to be linked to known species and enabling access to the biological information associated with those species. Environmental DNA surveys are becoming more common in New Zealand for quantifying ecological communities, assessing environmental states, and for biosecurity surveillance (Holdaway et al. 2017; Bulman et al. 2018; Dopheide et al. 2019; Watts et al. 2019; Dibattista et al. 2024; Wilkinson et al. 2024). However, in many instances, the interpretation of these data is limited by a lack of species‐level taxonomic identifications (Bird et al. 2024).

Reference libraries are essential for DNA barcoding, and their continued population with DNA barcode data from taxonomically focused studies is imperative (Collins et al. 2026). There are numerous national‐scale efforts to DNA barcode faunas of different countries (Geiger et al. 2016; deWaard et al. 2019; Weigand et al. 2019; Price et al. 2020; García‐Ruilova et al. 2026). Furthermore, recent advances in DNA extraction and sequencing technologies have improved the efficiency of DNA barcode generation (Meier et al. 2016; Dopheide et al. 2025), making the completion of national‐scale DNA barcode libraries an increasingly realistic goal. In New Zealand, there is currently no national strategy, but there have been several DNA barcoding efforts focused on specific insect orders or families, including Hemiptera (Podmore et al. 2015; Martoni et al. 2018; Podmore et al. 2019), grasshoppers (Orthoptera) (Trewick 2008), mosquitoes (Diptera) (Wu et al. 2023), weevils (Coleoptera) (Leschen et al. 2022), moths (Lepidoptera) (Langhoff et al. 2009; Wöger et al. 2020), caddisflies (Hogg et al. 2009) and Hymenoptera (Ward 2024). At least one study has targeted a broad range of invertebrates for DNA barcoding at a single geographic location (Dopheide et al. 2019) and many evolutionary studies have included the COI gene as a phylogenetic or phylogeographic marker, therefore generating additional DNA barcode data. However, researchers and practitioners are increasingly advocating the need to develop a national reference DNA barcode database for New Zealand (Bunce and Freeth 2022; Dhami et al. 2025).

One of the first steps in developing a national strategy is a stock‐take of existing DNA barcode taxonomic coverage (Kgatla et al. 2026), followed by a co‐ordinated plan to gap‐fill. Here, we assess the prospects for compiling a complete DNA barcode reference library for the New Zealand insect fauna. In this context, a ‘complete' DNA barcode reference library means that every insect species is represented by at least one DNA barcode. This includes described and undescribed species, acknowledging that in the case of undescribed species, it is not possible to be completely confident as to when the end goal is reached. Accordingly, the goals of this study are to: (1) determine the proportion and taxonomic composition of the New Zealand insect fauna that has been barcoded for both described and undescribed species; (2) evaluate taxonomic, geographic and elevational biases in these data; (3) assess the identification methods used to assign taxonomic names to DNA barcodes; and (4) propose recommendations for completing the DNA barcode library for the New Zealand insect fauna.

## 2 Materials and Methods

### 2.1 Assessment of DNA Barcode Coverage From Exploration of Databases

We queried the New Zealand Organisms Register (NZOR 2012) to retrieve a list of insect species names that are labelled as current and present in New Zealand, excluding synonyms and entities listed only to genus. The names in NZOR are derived from the initial lists of names from MacFarlane et al. (2010), which are regularly updated soon after publication of new names or changes to nomenclature for terrestrial invertebrates as part of ongoing efforts to enhance Nationally Significant Collections and Databases. The NZOR name list excludes undescribed species and so cannot be used as an estimate of true species diversity. To assess DNA barcode coverage of undescribed species, we used species richness values for each insect order reported previously (MacFarlane et al. 2010) which were based on expert knowledge of the local insect fauna. Although the number of described species has increased since the publication of the 2010 lists, a substantial proportion of these additions reflect the formal description of taxa previously recognised as undescribed. Consequently, this increase is unlikely to have significantly altered the estimated total species richness.

We downloaded all Insecta DNA barcodes and associated metadata, returned via search query: tax  =  ‘Insecta’ AND geo  =  ‘New Zealand’, from the Barcode of Life Database (BOLD; accessed 13‐02‐2026) (Ratnasingham and Hebert 2007). Setting the geo field to ‘New Zealand' will recover all DNA barcode records derived from specimens that were collected from New Zealand but will exclude New Zealand species collected from other countries. We limited these barcodes to COI‐5P records. Because DNA barcodes are also deposited in GenBank, we downloaded the LONGEST_NUC_GB265_CO1 component of the MIDORI2 database (version GenBank265_2025‐03‐08) and extracted any barcodes for New Zealand insect species names that were not already represented among the BOLD barcodes and with sequence lengths ≥500 bp. We note that these latter barcodes lack the metadata associated with those obtained from BOLD.

We matched binomial species names in NZOR to species names from the BOLD and MIDORI2 Insecta DNA barcodes and assessed the numbers of species with and without DNA barcodes relative to biostatus in each insect order. We compared the number of BOLD BINs from each insect order to the total number of described, known undescribed, and estimated undescribed species from MacFarlane et al. 2010, expressed as a percentage. This approach assumes that there is a strong correlation between BIN and species. We also examined DNA barcoding disparities between families within Coleoptera, as this is the most diverse insect order in New Zealand and, therefore, a high priority for DNA barcoding.

We analysed the metadata associated with New Zealand Insecta DNA barcodes from BOLD. We compared the relative proportions of different methods for identifying specimens or DNA barcodes and the lowest taxonomic rank that each DNA barcode was identified to. DNA barcode records were split into bioinformatic or morphological identification methods if the “identification_method” in the json file included keywords “bin”, “DNA”, “tree”, “BOLD”, “molecular”, “barcoding” or “data release”. DNA barcode records were assigned to the morphological category if they included the keywords “morph”, “examination”, “examined”, “examined material”, “cleared genitalia”, “det label” or included the name of a known taxonomic specialist. Geographic coordinates associated with these BOLD barcodes were used to visualise spatial sampling patterns and elevational coverage. Many of these barcodes lacked georeferences because they were originally sourced from Genbank, and for these, we added georeferences from corresponding records in GBIF. Further inspection of BOLD metadata revealed many occurrences of default or placeholder coordinate rather than a true collection locality. Records assigned to such coordinates were excluded prior to analysis, and records lacking geographic coordinates were excluded from all spatial‐ and elevation‐based analyses, yielding a single filtered dataset. To visualise geographic sampling density, records were aggregated by unique coordinate pairs and plotted as a bubble map, with point size proportional to the number of DNA barcodes per location. Elevational sampling bias was assessed by plotting the distribution of estimated elevations as histograms based on the filtered dataset. Where elevation was not included in the BOLD metadata, elevation for each record was extracted from a digital elevation model (DEM; SRTM 90 m) using the metadata georeferences. For elevation analysis, an additional filtering step was undertaken, where we removed records with implausible elevation values (e.g., negative elevations), and only records with valid, non‐missing estimated elevations were retained. All analyses and plots were performed/created in R version 4.4.0 (R Core Team 2023).

## 3 Results

### 3.1 Taxonomic Coverage of DNA Barcodes From New Zealand Insects

As of May 2025, NZOR contained a total of 13,959 unique insect species names that are labelled as current and present in New Zealand, of which 11,977 are native, 1701 are exotic and 281 are of unknown biostatus (Table 1). The number of described species per order ranges from 1 in Mecoptera and Megaloptera to 5778 in Coleoptera (Table 1).

**TABLE 1:** Numbers of described native and exotic species present in New Zealand from each insect order (from NZOR) and percentages with DNA barcodes (from BOLD and MIDORI2).

Order	Number of species	Species with barcodes	Number of native species	Native species with barcodes	Number of exotic species	Exotic species with barcodes
Archaeognatha	2	0 (0%)	2	0 (0%)	0	—
Blattodea	39	20 (51%)	25	11 (44%)	14	9 (64%)
Coleoptera	5778	611 (11%)	5220	343 (7%)	482	262 (54%)
Dermaptera	22	7 (32%)	13	1 (8%)	9	6 (67%)
Diptera	2798	274 (10%)	2532	131 (5%)	198	117 (59%)
Ephemeroptera	52	36 (69%)	52	36 (69%)	0	—
Hemiptera	1146	263 (23%)	847	76 (9%)	292	185 (63%)
Hymenoptera	1138	325 (29%)	832	139 (17%)	302	183 (61%)
Isoptera	9	4 (44%)	4	1 (25%)	5	3 (60%)
Lepidoptera	1838	519 (28%)	1524	319 (21%)	212	167 (79%)
Mantodea	2	2 (100%)	1	1 (100%)	1	1 (100%)
Mecoptera	1	1 (100%)	1	1 (100%)	0	—
Megaloptera	1	1 (100%)	1	1 (100%)	0	—
Neuroptera	15	7 (47%)	7	2 (29%)	8	5 (62%)
Odonata	20	11 (55%)	12	7 (58%)	5	3 (60%)
Orthoptera	149	74 (50%)	132	63 (48%)	7	1 (14%)
Phasmatodea	26	17 (65%)	26	17 (65%)	0	—
Plecoptera	107	71 (66%)	107	71 (66%)	0	—
Psocodea	417	64 (15%)	315	32 (10%)	92	29 (32%)
Siphonaptera	28	8 (29%)	16	0 (0%)	11	8 (73%)
Strepsiptera	3	0 (0%)	3	0 (0%)	0	—
Thysanoptera	133	32 (24%)	72	2 (3%)	61	30 (49%)
Zygentoma	6	0 (0%)	4	0 (0%)	2	0 (0%)
Trichoptera	229	175 (76%)	229	175 (76%)	0	—
Total	13,959	2522 (18%)	11,977	1429 (12%)	1701	1009 (59%)

**FIGURE 1:**
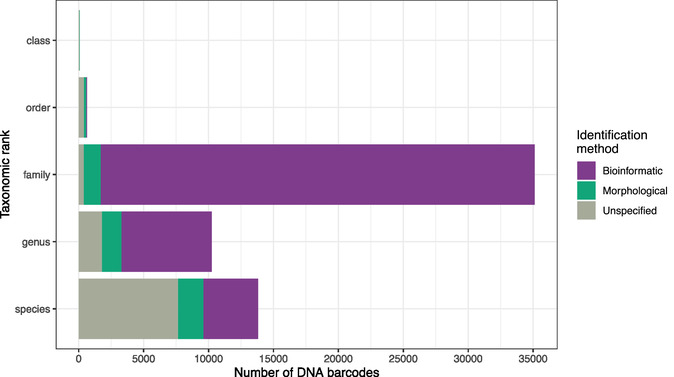
Stacked bar chart showing the number of barcode records identified to class, order, family, genus and species as well as the method of identification for New Zealand insect COI DNA barcode records from BOLD. Just two records are identified to class, both by morphological methods.

The BOLD database contained a total of 49,661 COI DNA barcodes tagged from New Zealand Insecta, with 0.1% identified only to class, 0.4% to order, 65% identified to family, 18.3% to genus and 16.2% identified to species (Figure 1), including 1293 unique species names. COI barcodes for a further 1140 species (mainly exotics) were recovered from MIDORI2. In total across both databases, one or more DNA barcodes were available for 2522 of the 13,959 insect species (18.1%), of which 1429 are native, 1009 are exotic, and 84 have unknown biostatus (Table 1). This corresponded to one or more COI DNA barcodes being found for 59% of exotic insect species but only 12% of native insect species (Table 1). Conversely, COI DNA barcodes were not available for a total of 11,437 insect species (82%), of which 10,548 are native, 692 are exotic, and 197 have unknown biostatus.

The proportion of native species with DNA barcodes varied extensively among insect orders. Some orders with extremely low diversity, such as Archaeognatha (only two described species; Mendes et al. 1994), lacked any DNA barcodes with species‐level identification. In contrast, some orders with relatively moderate levels of diversity, such as Trichoptera (229 described native species; MacFarlane et al. 2010), had 76% of named species with a DNA barcode (Table 1). Similarly, two other moderate‐diversity aquatic insect orders, Ephemeroptera and Plecoptera, also have relatively high DNA barcode coverage of 69% and 66%, respectively. The native species‐rich insect orders of Hemiptera, Hymenoptera, Lepidoptera, Diptera and Coleoptera had varying levels of barcode coverage: 9%, 17%, 21%, 5% and 7%, respectively (Table 1). Among these, Coleoptera is notable as having the highest number of described native species (5220) but a very low percentage with DNA barcodes (Table 1). The only orders with complete barcode coverage were Mantodea, Mecoptera and Megaloptera, each of which have only a single native described species (Table 1).

We compared the estimated total number of native insect species from each order with the number of insect BINs tagged as geo  =  New Zealand on BOLD (Table 2). We used this comparison to estimate the proportion of the insect species, including undescribed species, in each order that is represented by a DNA barcode. For example, Trichoptera are estimated to have 304 species, and 276 BINs are allocated to this order, suggesting coverage of 91% (Table 2). Based on this metric, the other two largely aquatic orders of Plecoptera and Ephemeroptera have 31% and 75% coverage, respectively (Table 2). Estimated coverage in other moderate‐diversity orders is lower; for example, Psocodea putatively has 469 species yet there are only 31 BINs (7% coverage) associated with this order (Table 2). The coverage of the high‐diversity orders Hemiptera, Hymenoptera, Lepidoptera, Diptera and Coleoptera is quite variable, ranging from 4% of Coleoptera species to 49% of Hymenoptera species (Table 2).

**TABLE 2:** Total numbers of described, known undescribed, and estimated unknown species (from MacFarlane et al. 2010, BOLD barcodes and BINs and ratios of BINs to species, for each insect order found in New Zealand).

Order	Described species	Known undescribed species	Estimated unknown species	Estimated Total species	BOLD barcodes	BOLD BINs	Ratio of BINs/total species
Archaeognatha	2	0	2	4	2	1	25%
Blattodea	35	9	5	49	46	5	10%
Coleoptera	5062	420	3000	8482	3587	351	4%
Dermaptera	21	1	3	25	12	3	12%
Diptera	2483	786	1640	4909	28,749	1135	23%
Ephemeroptera	48	3	10	61	236	46	75%
Hemiptera	1079	78	440	1597	3986	178	11%
Hymenoptera	721	934	500	2155	7556	1065	49%
Isoptera	9	0	0	9	0	0	—
Lepidoptera	1686	14	100	1800	2346	439	24%
Mantodea	2	0	0	2	6	2	100%
Mecoptera	1	0	0	1	1	1	100%
Megaloptera	1	0	0	1	169	2	200%
Neuroptera	14	0	0	14	60	9	64%
Odonata	17	0	0	17	8	6	35%
Orthoptera	116	69	5	190	26	10	5%
Phasmatodea	22	7	7	36	4	2	6%
Plecoptera	99	21	20	140	249	43	31%
Psocodea	402	17	50	469	913	31	7%
Siphonaptera	28	0	5	33	0	0	—
Strepsiptera	2	1	2	5	0	0	—
Thysanoptera	122	1	10	133	134	24	18%
Trichoptera	228	26	50	304	1,534	276	91%
Zygentoma	3	8	8	19	0	0	—
Total	12,203	2395	5857	20,455	49,624	3629	18%

Examination of the BOLD metadata associated with all DNA barcodes from New Zealand insects indicates that most specimens were identified using bioinformatic methods—mostly BIN, BLAST or phylogenetic approaches—and only a small proportion of DNA barcodes were identified to species (Figure 1). The relative proportions of identification method vary depending on whether the DNA barcode is identified to species or only to a higher taxonomic rank. For example, most DNA barcodes identified to family only used bioinformatic approaches, whereas DNA barcodes identified to species do not specify the identification method used (Figure 1).

**FIGURE 2:**
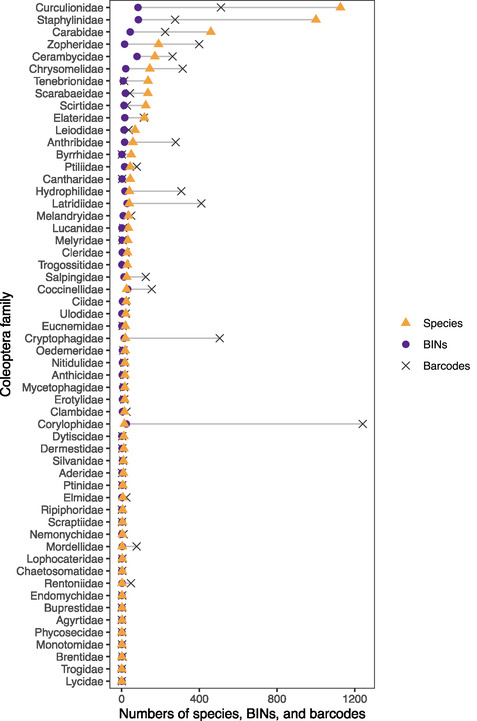
Numbers of available DNA barcodes, estimated BINs, and described species from families of Coleoptera with records of being present in New Zealand.

We also examined the number of species, DNA barcodes and BINs from Coleoptera families, which showed extreme variation in species richness, ranging from one to over 1000 species per family (Figure 2). The family with the most DNA barcodes (≈700) was Corylophidae, with only 14 described species and 20 BINs (Figure 2). The most speciose family, Curculionidae (weevils), had the largest disparity between numbers of species (1126) and BINs (54), despite having more BINs than any other family. Some relatively diverse Coleoptera families, such as Scirtidae, Scarabaeidae and Tenebrionidae, had very few DNA barcodes and BINs (Figure 2).

**FIGURE 3:**
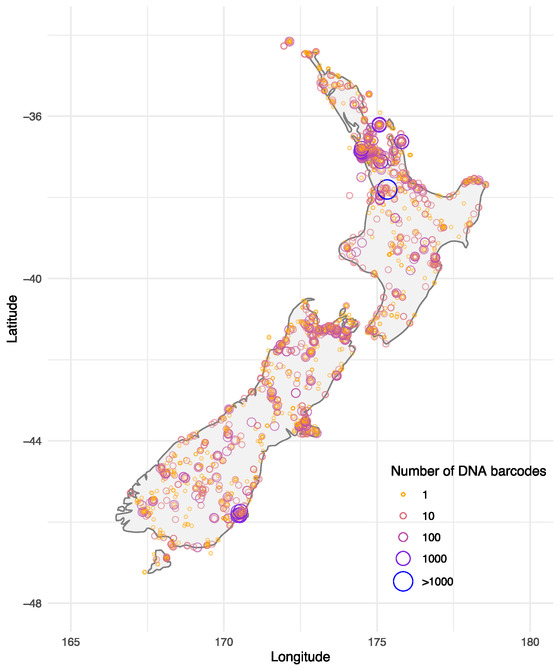
Geographic distribution of DNA barcode records for New Zealand insects for which georeferenced data are available. Bubble size is proportional to the number of DNA barcodes at each site. Points that appear to fall in the ocean are due to inaccuracies in georeferencing by data providers.

**FIGURE 4:**
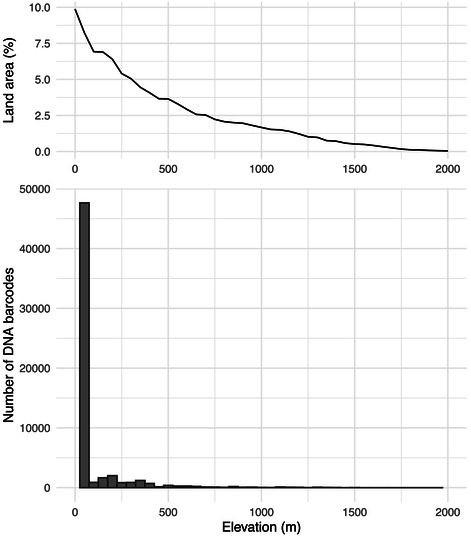
Bar chart showing the number of DNA barcodes binned by elevation that corresponding specimens were collected from. The line chart shows the percentage of New Zealand land area at different elevations.

We were able to obtain georeferences corresponding to collection locations for 46,657 out of 49,661 (94%) DNA barcode records and observed that the geographic distribution of DNA barcodes is highly unequal (Figure 3). Large numbers of DNA barcodes are from sampling sites in the Auckland, Waikato, Nelson, Christchurch and Dunedin regions. The elevational distribution of DNA barcodes is also highly biased towards low‐altitude locations (Figure 4). Very few DNA barcodes are from specimens collected above 500 m above sea level in elevation, whereas 97.8% of records were from lower than 500 m, in contrast to the land area of New Zealand (Figure 4). Only 0.3% of records were from sites above 1000 m, which is slightly below the tree line for much of the South Island (Case and Buckley 2015).

## 4 Discussion

Our analysis of online genetic databases shows that modest progress has been made towards a complete DNA barcode inventory of the New Zealand insect fauna, despite the lack of national‐level coordination and dedicated funding. Our analyses also indicate some concerns with current DNA barcode data, namely, a lack of clarity around specimen identification methodology, an over‐reliance on bioinformatic approaches to identification and highly biased taxonomic, geographic and elevational coverage. These biases and methodological concerns need to be addressed in future sampling. However, our results suggest that far from being an unachievable goal, a complete barcode library of New Zealand insects is within reach given a coordinated and strategic approach to taxon and specimen sampling.

### 4.1 Status of Current New Zealand Insect DNA Barcode Data

A modest start has been made to DNA barcoding the New Zealand insect fauna, with approximately 19% of described native and introduced insect species represented in DNA barcode databases. Furthermore, the ratio of BINs to estimated total number of insect species in New Zealand was 24%, suggesting that perhaps one‐quarter of New Zealand insect species are represented by a DNA barcode. However, most DNA barcodes available in online databases such as BOLD are identified to a taxonomic rank higher than that of the species, which limits their scientific utility. This finding agrees with analysis of DNA barcode records in BOLD from all taxa, where most records lack species‐level identification (Dhami et al. 2025).

New Zealand DNA barcode coverage is highly unequal among insect orders, with the hyperdiverse orders particularly poorly covered, especially Diptera and Coleoptera. However, even some low‐diversity orders have relatively low DNA barcode coverage of described species, one example being Archaeognatha—where several DNA barcodes are available, but none are attributed to the two described New Zealand species in this order (Mendes et al. 1994). On the other hand, DNA barcode coverage of the moderately diverse aquatic insect orders was particularly good. For example, the ratio of BINs to estimated species reached 91% in Trichoptera. This high level of coverage likely reflects four factors: (i) the moderate species diversity of these orders (17, 61, 140 and 304 estimated species in Odonata, Ephemeroptera, Plecoptera and Trichoptera, respectively); (ii) their relatively recent taxonomic focus (McLellan 1993; Ward 1995; Foster et al. 2020); (iii) their inclusion in many phylogenetic (Nolan et al. 2007; McCulloch et al. 2009; Winterbourn et al. 2017), DNA barcoding (Hogg et al. 2009) and eDNA (Wilkinson et al. 2024) studies; and, most importantly, (iv) a well‐established national biodiversity monitoring framework for freshwater invertebrate biodiversity (Stark 1993).

Within insect orders, the barcode coverage is also heavily biased. For example, the Coleoptera family with the greatest number of DNA barcodes was Corylophidae, which contains approximately 14 described species in New Zealand (MacFarlane et al. 2010). On the other hand, some moderate‐to‐high‐diversity families, such as Tenebrionidae, Scarabaeinae and Scirtidae, were represented by very few DNA barcodes. This pattern indicates opportunistic sampling rather than systematic representation of evolutionary or taxonomic richness. Future sampling needs to consider within‐order biases as well as between‐order biases in DNA barcode coverage.

The geographic distribution of DNA barcode data was highly biased towards populated regions of New Zealand. This bias is partly attributable to the substantial number of DNA barcodes generated from insect specimens collected using Malaise traps in the Waikato region (Seymour et al. 2024). However, sampling effort is also clearly concentrated in the major population centres of Auckland, Christchurch and Dunedin, as well as in the Nelson region. Nelson has historically been a focal area for insect collecting due to its high habitat heterogeneity within a relatively small geographic area, well‐documented endemism across plants and animals (Millar et al. 2017; Taylor‐Smith et al. 2020), and until the 1970s, was the location of the New Zealand Arthropod Collection. The signal of individual studies is also seen in the country‐wide data, for example, in the biodiversity survey of Hauturu (Little Barrier Island, near Auckland), where large numbers of invertebrates were DNA barcoded by Dopheide et al. (2019). While our DNA barcode coverage map shows sampling from across New Zealand, many areas are sparsely covered or have relatively small numbers of DNA barcodes, meaning that the unique insect diversity in these areas is very poorly sampled. Indeed, elevational representation of DNA barcode records is strongly skewed toward lowland sites, and much of the data from higher‐altitude specimens result from phylogenetic studies of alpine species (Trewick et al. 2000; Chinn and Gemmell 2004; Buckley and Simon 2007), rather than deliberate efforts to systematically sample alpine insect taxonomic diversity. Therefore, much of New Zealand's rich biogeographic and altitudinal diversity of insect fauna (Dumbleton 1970; Taylor‐Smith et al. 2020; Buckley et al. 2024) is not included in DNA databases, and future sampling needs to address these biases. We note that our estimates of biased barcode coverage per order and geographic/elevation apply only to BOLD‐derived barcodes, due to an absence of BIN information and relevant metadata associated with GenBank‐derived MIDORI2 sequences. However, most of the MIDORI2‐derived barcodes were for exotic species, indicating that these patterns are likely robust for native species.

The high proportion of DNA barcodes that lack information on identification methods or use a bioinformatic approach is concerning. Accurate specimen identifications are essential for DNA barcodes derived from a reference library if the library is intended to support authoritative identifications. Identification errors in the reference library have the potential to propagate through the library as new DNA barcodes are added and assigned identifications only bioinformatically. The lack of metadata on identification methods makes it impossible to ascertain the reliability of a given DNA barcode. We recommend that, prior to DNA barcode sequencing, insect specimens be accurately identified using a method that is appropriate for the taxon and the identification method should be explicit so that it is repeatable and verifiable (Vink et al. 2012). We acknowledge that specimens housed in collections will usually not record the method of identification, but the name of the determiner is often recorded as label data or in a specimen database, potentially giving confidence in the identification. We also recommend that where expertise is available (and the specimens still exist), a concerted effort is made to assign species names to the thousands of specimens that have been previously DNA barcoded but were only identified bioinformatically or only to a higher taxonomic rank such as family. Doing this would dramatically improve the scientific value of the data.

### 4.2 How to Complete DNA Barcoding the New Zealand Insect Fauna

Given these gaps and biases, a strategic approach to complete the New Zealand insect DNA barcode library must be developed. The simplest approach is arguably to start with the described species, as specimens are available for virtually all these species, and tracking progress is relatively straightforward given the availability of species name lists. There are 11,437 species lacking DNA barcodes, and breaking these down by order offers a further way of approaching DNA barcoding. For example, 14 of the 24 insect orders in New Zealand have fewer than 100 described species each (MacFarlane et al. 2010), of which many species have already been sequenced. Sequencing the remaining 149 species within these orders would achieve complete barcode coverage for them, thereby capturing a substantial proportion of New Zealand's insect phylogenetic and taxonomic diversity.

Completing the DNA barcode library for the hyper‐diverse orders Hemiptera, Hymenoptera, Coleoptera, Diptera and Lepidoptera will be more challenging. There are several complementary ways to prioritise taxon sampling within these hyper‐diverse orders. For example, initially, one or more species from each genus can be sampled, followed by subsequent complete species‐level sampling. This approach at least yields an initial framework that covers all genera within an order, and therefore, a large component of evolutionary diversity. Another approach is to prioritise families of greatest interest for biosecurity, conservation or environmental monitoring. Targeting rare species can be justified to improve the identification of threatened species, and thereby, enhance knowledge of their distribution and abundance, particularly when combined with geographically extensive bulk sampling or eDNA surveys. Targeting common species can also be justified because these species are more likely to be represented in ecological surveys (Srivathsan et al. 2023) and have a disproportionate effect on ecological function (Gaston 2011). Being able to recognise that the same species are repeatedly sampled across studies and being able to connect the specimens to a taxonomic name (if available) helps immensely with the biological interpretation.

The second goal of generating DNA barcodes for the estimated 2395 and 5857 known undescribed and unknown undescribed New Zealand insect species is more challenging. This involves barcoding both known undescribed species, i.e., specimens in collections identified as belonging to an undescribed species, and unknown species that are predicted to exist but have not yet been collected (MacFarlane et al. 2010). The former are represented by unsorted or unidentified specimens in collections and are, in principle, available for DNA sequencing. However, many additional species remain to be discovered and collected across New Zealand. Obtaining material for these unsampled species is perhaps the most difficult aspect, requiring field surveys with bulk‐collection methods, such as Malaise and pitfall traps, together with targeted sampling of specific microhabitats (e.g., leaf litter and dead wood sifting and light trapping) to capture a broad range of insect hyperdiversity. Again, this will require dedicated taxonomic expertise to identify specimens or determine that specimens represent undescribed species. The result of this process—i.e., bulk collecting, sorting samples and DNA barcoding—will be many MOTUs or BINs that cannot be attached to a species name. While initially of less utility than DNA barcodes that are attached to species names, these data still have value as measurable units for exploring diversity in hyperdiverse groups that are poorly known taxonomically (Hartop et al. 2024). Such MOTUs and BINs can be used as categories for studying biodiversity, pending later taxonomic description (Buckley 2025; Meier, Lawniczak, et al. 2025). Ultimately, once a ‘taxonomic backbone’ is established from targeted sampling of described species, MOTUs and BINs representing undescribed species can be integrated into that framework. Data collection and analysis pipelines are rapidly developing to achieve this goal (Srivathsan et al. 2019; Wührl et al. 2022; Meier, Srivathsan, et al. 2025). Monitoring progress to the goal of sampling undescribed species is a relatively straightforward process: as specimens and knowledge accumulate, rarefaction analyses can be used to assess convergence to the end point. Finally, it is important to ensure that eventually these DNA barcodes are attached to formal species names, and this will require increased funding for taxonomy (Leschen et al. 2016; Buckley 2025).

### 4.3 Methodological Considerations

For those described species represented in collections by specimens collected decades ago, ‘museomic' approaches will likely be required to generate DNA barcodes (Fong et al. 2023; Santos et al. 2023). This involves a combination of specialised DNA extraction methods (Holmquist et al. 2025) and genome skimming for recovery of DNA barcodes (Ferrari et al. 2023) as well as short overlapping polymerase chain reaction (PCR) fragments. Genome skimming methods are, however, currently expensive relative to PCR, and it is likely that in the short term, at least, the former will remain limited to cases where DNA is extremely fragmented in very old specimens.

A DNA barcode reference library and associated specimen and DNA voucher management workflows should be future‐proofed for ongoing changes in DNA sequencing technology and cost. A price point for DNA sequencing will eventually be reached where it will make more sense to sequence whole genomes, even if they are highly fragmented in the case of specimens from historical collections. Whether low‐coverage or high‐quality, genomes provide much richer biological information than DNA barcodes alone, and having a ‘backbone’ of genomic information incorporated into the reference library will enhance its scientific value. Furthermore, the analysis of short fragments (i.e., k‐mers) of DNA sequences from across a genome can yield strong signals for species identification (de Medeiros et al. 2025) and phylogenetic relationships (Zhang and Nielsen 2026). DNA extractions are stored in such a way as to facilitate DNA sequencing with future technologies. This is especially important for species represented by very few specimens, where repeatedly sampling tissues and damaging the specimen are undesirable. There are several global initiatives that are generating whole genome datasets for entire faunas (Lewin et al. 2018; Darwin Tree of Life Consortium 2022), and these will be useful models for genomic surveys of New Zealand insects.

A critical step in planning the completion of the New Zealand DNA barcode library is determining the degree of intraspecific sampling. Many New Zealand terrestrial invertebrate species show very high levels of mitochondrial DNA diversity (Trewick et al. 2000; Boyer et al. 2007; Marske et al. 2011). Although sequencing multiple individuals per species significantly increases the cost of DNA barcoding and will slow down progress towards complete coverage of all species in the database; it is critical for several reasons. First, species identification through DNA barcoding will be more accurate when the reference database has data from multiple specimens, ideally sampled in such a way as to capture intraspecific diversity (Luo et al. 2015; Phillips et al. 2022). Second, having data from multiple individuals allows clustering into BINs (Ratnasingham and Hebert 2013; Collins et al. 2026) or MOTUs (Srivathsan et al. 2019; Hartop et al. 2022). Therefore, we recommend sampling multiple individuals within a species. Where specimens are known only from the holotype, a risk assessment needs to balance the scientific value of the DNA barcode against the integrity of the holotype, and wherever possible, non‐destructive DNA extraction methods should be employed (Castalanelli et al. 2010) or a small amount of tissue should be drawn from a part of the body deemed less important for morphological identification (Mutanen et al. 2015).

It is also essential that DNA barcodes are obtained from specimens that are accessioned into public biological collections and that all DNA barcoded specimens have accessible digitised metadata. In devising a DNA barcoding strategy, an accurate database of accepted taxonomic names is also important to ensure that specimen sampling follows the currently accepted classification (Geiger et al. 2016). Using outdated or static lists of species names to guide sampling will lead to gaps in the DNA barcode library if more recently published names are omitted. Finally, we note that management, governance and access of any DNA barcode library will need to address ethical concerns including Indigenous data sovereignty (Dhami et al. 2025; Adams et al. 2026; Frankiewicz et al. 2026).

## 5 Conclusion and Recommendations

DNA barcode data from New Zealand insects exhibit several biases. Nevertheless, a complete DNA reference library is feasible. Completion of the reference library requires a systematic approach and coordinated infrastructure. This needs to involve the integration of taxonomic expertise and collections, curation of digital data, field surveys and DNA sequencing. We recommend the following strategy to build a complete DNA barcode database for New Zealand insects.

### 5.1 Taxonomic Resources

1. Increase support for expert taxonomic identification so that DNA barcodes are accurately linked to species names, with the identification method or source recorded in metadata.
2. Ensure enduring support for taxonomic collections, which offer a rich source of reliably identified specimens for DNA barcoding.

### 5.2 Maintain and Update Digital Records

1. Increase support for the maintenance of national checklists of taxonomic names, which are a baseline that underpins conservation and biosecurity. This includes maintenance of the names (ongoing data entry and updates) and checks to ensure the classification between databases (e.g., NZOR, BiotaNZ and BOLD) is consistent.
2. Acknowledge the efforts needed for digital curation of records within the DNA reference library. Such activities improve accuracy, reduce errors and give end‐users confidence in the reference library.

### 5.3 Field Surveys

1. Increase field surveys to reduce biases in the current dataset and more accurately document the diversity of the New Zealand insect fauna.
2. Establish a coordinated network of bulk collection sites spanning the geographic, ecological, and elevational range of New Zealand insect distributions.

### 5.4 Prioritisation of Insect Taxa for DNA Barcoding

1. Prioritise complete representation of 10,548 described native species that currently lack DNA barcodes before proceeding to complete coverage of undescribed species.
2. Complete DNA barcode coverage of the 149 described species from low‐ to moderate‐diversity insect orders that currently lack DNA barcodes.
3. Complete species‐level DNA barcode coverage of described species from the high‐diversity insect orders Coleoptera, Diptera, Hemiptera, Hymenoptera and Lepidoptera. Start by completing generic level sampling to build a taxonomic backbone before completing species level sampling.
4. Use specimens generated through bulk sampling, following expert identification, to close remaining gaps in barcode coverage of described species. Cluster specimens that cannot be confidently identified or that represent undescribed species into MOTUs or BINs.
5. Use rarefaction analyses to monitor progress towards completing DNA barcode coverage of the undescribed New Zealand insect fauna.

## Funding

This study was supported by Strategic Science Investment Funding (Infrastructure) and a Catalyst Seeding grant (CSG‐LCR2401) from the Ministry for Business, Innovation and Employment.

## Ethics Statement

The authors have nothing to report.

## Conflicts of Interest

The authors report there are no competing interests to declare.

## Data Availability

The data analysed here and associated scripts are available at the MWLR Datastore (https://doi.org/10.7931/cr73‐ap59).
